# Healthy worker effect phenomenon

**DOI:** 10.4103/0019-5278.55123

**Published:** 2009-08

**Authors:** Divyang Shah

**Keywords:** Healthy Worker Effect, occupational epidemiology, selection bias

## Abstract

The Healthy Worker Effect (HWE) phenomenon has been under debate since some years. Some epidemiologists regard HWE as an ordinary method problem while others consider it a field of science by itself. This article gives definitions of HWE explained with historical background; discusses factors affecting it and suggests methods to minimize problems associated with it.

## INTRODUCTION

The Healthy Worker Effect (HWE) phenomenon has been under debate since a few years. Some epidemiologists consider HWE an ordinary method problem while others consider it a field of Science by itself. In this article I shall explain various definitions of HWE with their historical background, necessary to understand the phenomena. I will also discuss factors affecting the phenomena and ways to deal with them.

## DEFINITIONS

“HWE is a phenomenon initially observed in studies of occupational diseases: Workers usually exhibit lower overall death rates than the general population because the severely ill and chronically disabled are ordinarily excluded from employment” – Last, 1995.[1]

Another definition by McMichael (1976)[2] who first gave it the name is: “HWE refers to the consistent tendency of the actively employed to have a more favorable mortality experience than the population at large.”

However, other occupational epidemiologists describe HWE as the reduction of mortality or morbidity of occupational cohorts when compared with the general population.

Let's try to understand it by an example.

Doll and coworkers[3] studed on gas workers exposed to carbonized coal. They measured standardized mortality rate (SMR i.e. mortality rates after eliminating possible effect of age differences in workers and general population) for groups of gas workers with different kinds of exposure. Following were the observations.

Mortality (SMR-, all causes) of gas workers compared with national experience

**Table:** 

Heavy Exposure	105
Intermediate	90
No Exposure	84

(Doll *et al*. 1965).

SMR is less than 100 in unexposed workers.

## HISTORICAL BACKGROUND

This phenomenon was first observed in 1885 when William Ogle[4] found mortality rate dependent on difficulty of occupation; some occupations repel and some others attract workers. In other words, the more vigorous occupations had a relatively lower mortality rate when compared with the death rate in occupations of an easier character or among the unemployed. Almost 100 years later, in 1974, McMichael coined the term HWE to describe this phenomena.[5] A year later, Goldsmith (1975)[6] pointed out that most industrially employed cohorts should be expected to have better life expectancy than unemployed persons. SMR close to unity (100) is used as an indication of absence or a low degree of HWE.

## IMPORTANCE OF HWE

Any occupational study looking for workers' health could potentially face this problem. It is a type of bias. The question is - Is it a serious bias? Most studies indicate that HWE will reduce the association between exposure and outcome by an average of 20-30%. Looking into the previous example of British gas workers:

**Table:** 

Heavy Exposure	Intermediate	No Exposure
105	90	84

SMR in non exposed worker is less than 100. So this reduction in SMR could be leading us to conclude that the condition among workers is good and no harmful effect was seen. This is not true. It may partially or completely mask excess mortality or morbidity caused by harmful exposure.

Trying to understand HWE was not easy considering the ongoing debate on its nature. Some scientists consider HWE a source of selection bias; others consider it confounding. A third group considers it a mix of both while some others look at it as a comparison problem.

### Selection Bias

“Error due to systematic differences in characteristics between those selected for study and those not.” The selection bias occurred from the initial choosing of workers (mainly healthy workers) and factors that influence the continuity of work such as leaving the work because of sickness. To put it in simple words, HWE refers to the initial hiring into workforce and subsequent factors which influence continuing employment.

### Confounding

“A situation in which a measure of the effect of an exposure on risk is distorted because of the association of exposure with other factor(s) that influence the outcome under study”.

The confounding factor is the (unmeasured) health status of the group of employees.

Going back to our example, GHS is associated with exposure. (Employment in industry and associated with outcome death).

A third school of thought considers HWE a confounder and selection bias since it is very difficult to differentiate between them. The last opinion was a comparison problem. According to Olli Miettinen;[7] the best reference for a population under study (as an example A) in a specific time is with the same population at the same time without the exposure – which is impossible.

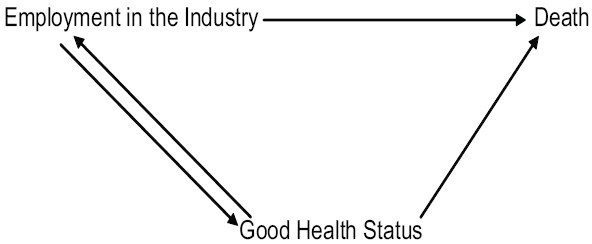

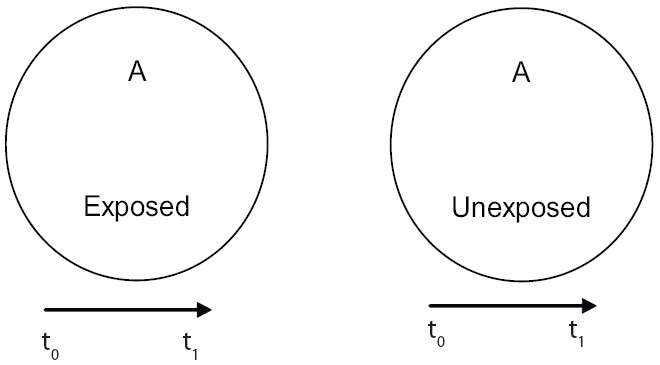





## COMPONENTS OF HWE

### Healthy Hire Effect

Employers have the right to reject certain persons for employment because of physical disabilities, or poor general health. An employer will exclude those obviously at high risk. Person selection may also be influenced by habits and physical conditions such as weight, smoking, or alcoholism. This effect will vary according to the labor situation, i.e. during period of labor shortages less fit workers could be included in the labor force whereas during periods of labor surplus employers can be choosy.

### Healthy Worker Survivor Effect

Workers who do not have strong motivation to work because of health problems do not present themselves for employment (self-selection). They generally change jobs frequently or retire early. They change their job for different reasons including health. The effect is reduced after 15 years of entry to the industry.

### Time-since-hire

The length of time the population has been followed. HWE is a characteristic of actively employed workers. Incomplete follow-up of the out-migrating section of the cohort could result in failure to track every individual to determine his vital status. Reduction in health status could occur without any relation to exposure.

Monson (1986)[8] divided the follow-up into two phases; a dynamic phase and a stable plateau. The dynamic phase is characterized by increased relative risk (RR) with years of follow-up. The RR becomes constant after some years of follow-up (plateau).

### Beneficial Effect of Work

Improved access to healthcare, routine disease screening and physical exercise is the beneficial effect of work. While there is a wide agreement on the first three factors, there is debate on the extent of the beneficial effect. Doll[3] considered low mortality a result of true benefit of work on health.

## FACTORS AFFECTING HWE

HWE is not constant. It varies depending on choice of comparison population. The factors affecting HWE also vary between studies.

### Time Related Factors Age at Hire

Workers with high age will be highly influenced by selective processes as the proportion of persons attaining the required level is likely to be smaller in the old age group.

### Example RR for all causes of mortality [Table 1] Age at risk

**Table 1:** RR for all causes of mortality

Age at Hire			
24-44	45-54	55-64	65-74
0.45	0.37	0.32	0.23

Fox and Collier (1976)[9]

The age at any point in follow-up that shows the outcome (death). Increasing age at risk will increase the period of follow-up and thus reduce the HWE.

### Example RR for all causes of mortality by age at risk [Table 2]

**Table 2:** RR for all causes of mortality by age at risk

Study		Age at Hire		
	<55	55-64	65-74	>= 75
Fox and Collier (1976)	0.64	0.79	0.96	0.60
McMichael et al. (1976)	0.81	0.89	0.95	1.13
Delzell and Monson (1981)[10]	0.80	0.90	0.90	1.00

### Duration of Employment

Increasing the duration of employment will increase the effect since many sick people will leave or shift to safer work.

### Socioeconomic Status

HWE is stronger in more qualified jobs. Professional workers demonstrate a stronger overall HWE. based on job classification; high socioeconomic status of work (white collar) has high healthy worker effects since it requires higher qualifications.

### Gender

Usually the effect is stronger for females than males.

## AVOIDING HWE

Many efforts have been made to minimize HWE. The most straightforward way is to avoid using general population as a reference group. Use active workers from another industry who do not have the same exposure. Another way to minimize HWE is to compare rates of health outcomes of interest between individuals with high exposure and those with low or no exposures. This is useful when the external reference group is not ideal. However, it is not likely that all occupational hazards pose gradient effects on human health (many industries show uniform exposure). One can also reduce HWE by starting the study after a latency of time e.g. one year, five years etc. where the HWE is high during this period. Another way to minimize HWE is to include the experience of every person who ever worked in a particular factory or industry

## CONCLUSION

HWE is caused by an inadequate reference group (i.e. comparison problem). If we find an ideal reference group then HWE will not exist. It is a complex, problem creating bias comprising of several factors and may be modified by a number of factors. It is not possible to make generalizations in a particular case of HWE.
